# Near-Infrared-Excitable Organic Ultralong Phosphorescence through Multiphoton Absorption

**DOI:** 10.34133/2020/2904928

**Published:** 2020-12-01

**Authors:** Ye Tao, Lele Tang, Qi Wei, Jibiao Jin, Wenbo Hu, Runfeng Chen, Qingqing Yang, Huanhuan Li, Ping Li, Guichuan Xing, Quli Fan, Chao Zheng, Wei Huang

**Affiliations:** ^1^Key Laboratory for Organic Electronics and Information Displays & Jiangsu Key Laboratory for Biosensors, Institute of Advanced Materials (IAM), Nanjing University of Posts & Telecommunications, 9 Wenyuan Road, Nanjing 210023, China; ^2^Joint Key Laboratory of the Ministry of Education, Institute of Applied Physics and Materials Engineering, University of Macau, Avenida da Universidade, Taipa, Macau 999078, China; ^3^Frontiers Science Center for Flexible Electronics (FSCFE), MIIT Key Laboratory of Flexible Electronics (KLoFE), Shaanxi Key Laboratory of Flexible Electronics, Xi'an Key Laboratory of Flexible Electronics, Xi'an Key Laboratory of Biomedical Materials & Engineering, Xi'an Institute of Flexible Electronics, Institute of Flexible Electronics (IFE), Northwestern Polytechnical University, Xi'an, 710072 Shaanxi, China

## Abstract

Organic ultralong room-temperature phosphorescence (OURTP) with a long-lived triplet excited state up to several seconds has triggered widespread research interests, but most OURTP materials are excited by only ultraviolet (UV) or blue light owing to their unique stabilized triplet- and solid-state emission feature. Here, we demonstrate that near-infrared- (NIR-) excitable OURTP molecules can be rationally designed by implanting intra/intermolecular charge transfer (CT) characteristics into H-aggregation to stimulate the efficient nonlinear multiphoton absorption (MPA). The resultant upconverted MPA-OURTP show ultralong lifetimes over 0.42 s and a phosphorescence quantum yield of ~37% under both UV and NIR light irradiation. Empowered by the extraordinary MPA-OURTP, novel applications including two-photon bioimaging, visual laser power detection and excitation, and lifetime multiplexing encryption devices were successfully realized. These discoveries illustrate not only a delicate design map for the construction of NIR-excitable OURTP materials but also insightful guidance for exploring OURTP-based nonlinear optoelectronic properties and applications.

## 1. Introduction

**Figure 1:**
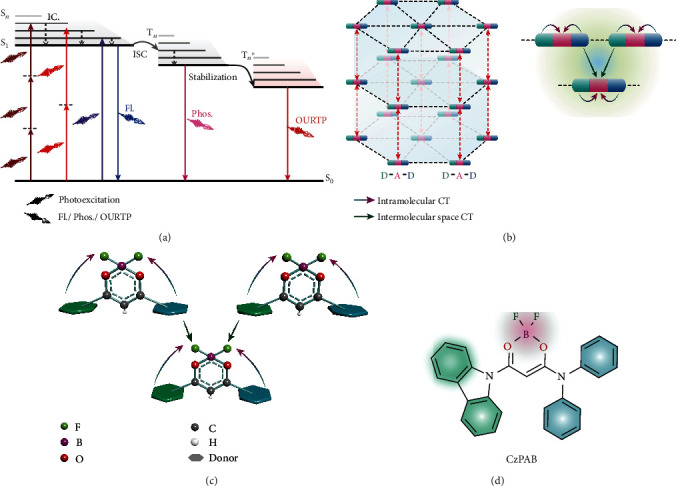
Molecular design strategy of MPA-OURTP materials. (a) Exciton transformation pathways of one-photon- (OPA), two-photon- (TPA), and three-photon- (3PA) triggered OURTP. The ground state (S_0_) molecule can be excited to S_*n*_ by absorption of one, two, or three photons and then fall to S_1_ through IC for fluorescence (Fl.). The triplet excited state (T_*n*_) can be populated from S_1_ via ISC, and the radiative decay of the lowest T_*n*_ (T_1_) leads to phosphorescence (Phos.), while by further stabilization for T_*n*_^∗^, OURTP is produced. (b) Design of MPA-OURTP molecules in a D-A-D architecture with strong and abundant in-plane (dashed black) and interlayer (red line) intermolecular interactions in crystal. (c, d) Schematic drawing of (c) MPA-OURTP molecules using a planar *π*-conjugation donor and a difluoroboron *β*-diketonate acceptor with synergistic effects of intramolecular CT (ICT) and intermolecular space CT (SCT) for MPA and (d) the molecular structure of the designed model compound of CzPAB.

Long-lived organic phosphorescence with lifetime over 0.1 s has shown great significance in both scientific understandings and technological applications ranging from anticounterfeiting [1–3], persistent light-emitting diodes [4], bioimaging [5, 6], and temperature sensing [7] to logic computing [8]. With various molecular design strategies including host-guest doping [9–12], H-aggregation [1, 13–15], crystallization [16–18], polymerization [19–21], and metal-organic framework coordination [22], a large number of organic ultralong room-temperature phosphorescence (OURTP) materials have been developed with lifetimes up to ~22.4 s and quantum efficiency over 40% under ambient conditions [7, 23]. However, compared to the abundant and vivid emission behaviors of OURTP showing blue, green, red, and white and even dynamically varied colors, most OURTP can only be excited by ultraviolet (UV) or blue light [24–26], owing to the intrinsic multiple exciton transformation features of OURTP (Figure 1(a)), where the photoexcited excitons on the lowest singlet excited state (S_1_) should be transformed to the triplet exciton through intersystem crossing (ISC) on the high-lying triplet excited state (T_*n*_) firstly, followed by internal conversion (IC) and triplet stabilization processes to form the stabilized T_*n*_ (T_*n*_^∗^) [24]. Therefore, the radiative decay of the lowest T_*n*_^∗^ (T_1_^∗^) for the OURTP emission is doomed to have very large Stokes shift (~150 nm) and inherently require high-energy UV and/or blue light excitation (Scheme S1). To reduce the Stokes shift and move the excitation wavelength to the visible range, rigid molecules with long conjugation lengths [27], direct triplet excited state absorption [14], and halogen/pseudohalogen atom incorporation have been proposed [28, 29], but the longest excitation wavelength is still shorter than 500 nm. Considering the phototoxicity of UV and blue light, it is urgent to explore the low-energy light-excitable OURTP.

Multiphoton absorption (MPA) is a nonlinear process in which a molecule can be excited from its ground state (S_0_) to the excited state by simultaneously absorbing two or more photons [30]. Therefore, the excitation wavelength can be significantly redshifted to even the near-infrared (NIR) range, if MPA is activated at large MPA cross section for the upconverted emission. Considerable success has been achieved in designing MPA-featured organic fluorophores [31, 32], phosphors [33], and thermally activated delayed fluorescence materials [34] in donor-*π*-acceptor (D-*π*-A) architectures with extended charge delocalization. However, it is notoriously challenging to develop the MPA-OURTP, owing to the intrinsic difficulties in simultaneously satisfying (i) the incorporation of strong charge transfer (CT) into an organic skeleton to enhance molecular dipole and conjugation for guaranteeing the large MPA cross section and highly efficient nonlinear optical behaviors [30, 35], (ii) the involvement of heteroatoms to confer efficient *n*‐*π*^∗^ transition for boosting ISC [1], and (iii) the formation of definitely ordered molecular aggregation in a solid state for stabilizing the triplet excitons and suppressing the nonradiative decays [24].

Here, we propose a rational design strategy by embedding intra- and intermolecular CT characters into H-aggregation in a quadrupolar D-A-D architecture for MPA-OURTP. Specifically, the synergistic effects of strong intramolecular CT (ICT) and intermolecular space CT (SCT) will increase MPA cross section for the upconverted emission, and the H-aggregation will stabilize the triplet excitons for OURTP. With this strategy, we choose the strong electron-withdrawing difluoroboron *β*-diketonate (BF_2_bdk) as the central acceptor moiety and two *π*-conjugation arylamines of carbazole and diamine as donor units. This design can not only motivate efficient ICT and SCT between arylamines and BF_2_bdk to boost MPA in both single molecular and aggregated states [36, 37] but also facilitate ISC by the inherent nonbonding *p* electrons of boron (B) and lone pair electrons of nitrogen (N) and fluorine (F) [24, 38]; meanwhile, various interlocked interactions empowered by the multiple heteroatom incorporation (Figures 1(b) and 1(c)) also result in the greatly suppressed nonradiative decay of the excited states for high luminescent efficiency; the implanted planar *π*-conjugation arylamine endows the construction of H-aggregation, which is crucial in the stabilization of triplet excitons for OURTP.

## 2. Results

### 2.1. Synthesis and Photoluminescence Properties

**Figure 2:**
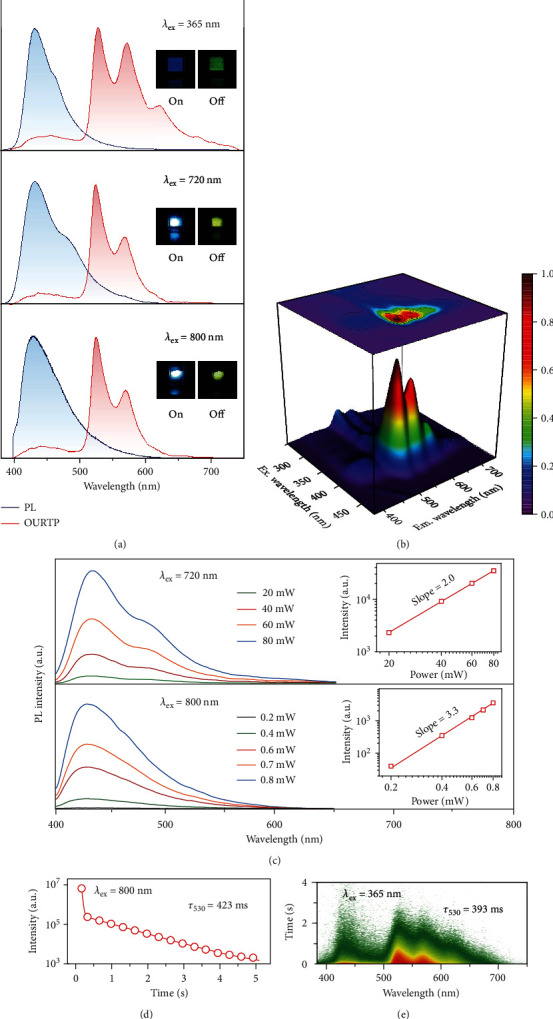
Photoluminescence properties of CzPAB powder under ambient conditions. (a) SSPL (blue) and OURTP (red) spectra excited by 365 nm UV light and 720 and 800 nm NIR lasers. Insets show the corresponding photographs on excitation (left) and removal (right) of the illumination light. (b) Excitation-OURTP emission mapping with a delay time of 25 ms. (c) SSPL spectra under different strengths of 720 (top) and 800 nm (bottom) NIR lasers with logarithmic plots of the integrated emission intensity versus the laser powers (insets). (d) OURTP lifetime decay profile at 530 nm excited by an 800 nm laser. (e) Transient emission decay image excited by 365 nm UV light.

As a proof of concept, difluoroboron 3-(9H-carbazol-9-yl)-3-oxo-N,N-diphenylpropanamide (CzPAB) was synthesized and systematically characterized (Figures S1–S9). Indeed, intense blue steady-state photoluminescence (SSPL) and yellowish OURTP emission from the CzPAB powder under ambient conditions can be excited by not only 365 nm UV light (Figure 2(a), top panel) but also NIR laser at 720 nm (Figure 2(a), middle panel) and 800 nm (Figure 2(a), bottom panel). Interestingly, the SSPL and OURTP spectra excited at 365, 720, and 800 nm are almost identical, exhibiting the main fluorescence band at 430 nm with nanosecond lifetime (Figure S10) and OURTP band at 530 nm with lifetime around 0.4 s (Figure S11 and Table S1). From the excitation-phosphorescence mapping, the OURTP of CzPAB can be excited from 240 to 420 nm (Figure 2(b) and Figure S12) with quite a low incident light intensity (20% Iris) and short irradiation duration time (<0.1 s) (Figure S13) as well as from the flashlight of a commercial mobile phone. The phosphorescence quantum yield of CzPAB powder at 365 nm excitation is as high as~37% under ambient conditions, which is among the best efficiencies of OURTP reported to date. To stimulate the nonlinear MPA process, 720 and 800 nm NIR lasers should be adopted and the higher laser power leads to stronger emissions (Figure 2(c)). From the power-dependent SSPL analyses, the integrated emission intensities are quadratic (slope ~2.0, Figure 2(c), inset top) and cubic (slope ~3.3, Figure 2(c), inset bottom) in response to the incident laser power, obviously verifying the two- and three-photon-excited luminescent feature at room temperature [30, 33]. Importantly, the MPA-OURTP exhibits nearly the same ultralong lifetime around 400 ms as the one-photon-excited OURTP by UV light (Figures 2(d) and 2(e) and Figure S11), suggesting the same decay process of the excitons photoexcited either linearly or nonlinearly.

### 2.2. Theoretical Investigations

**Figure 3:**
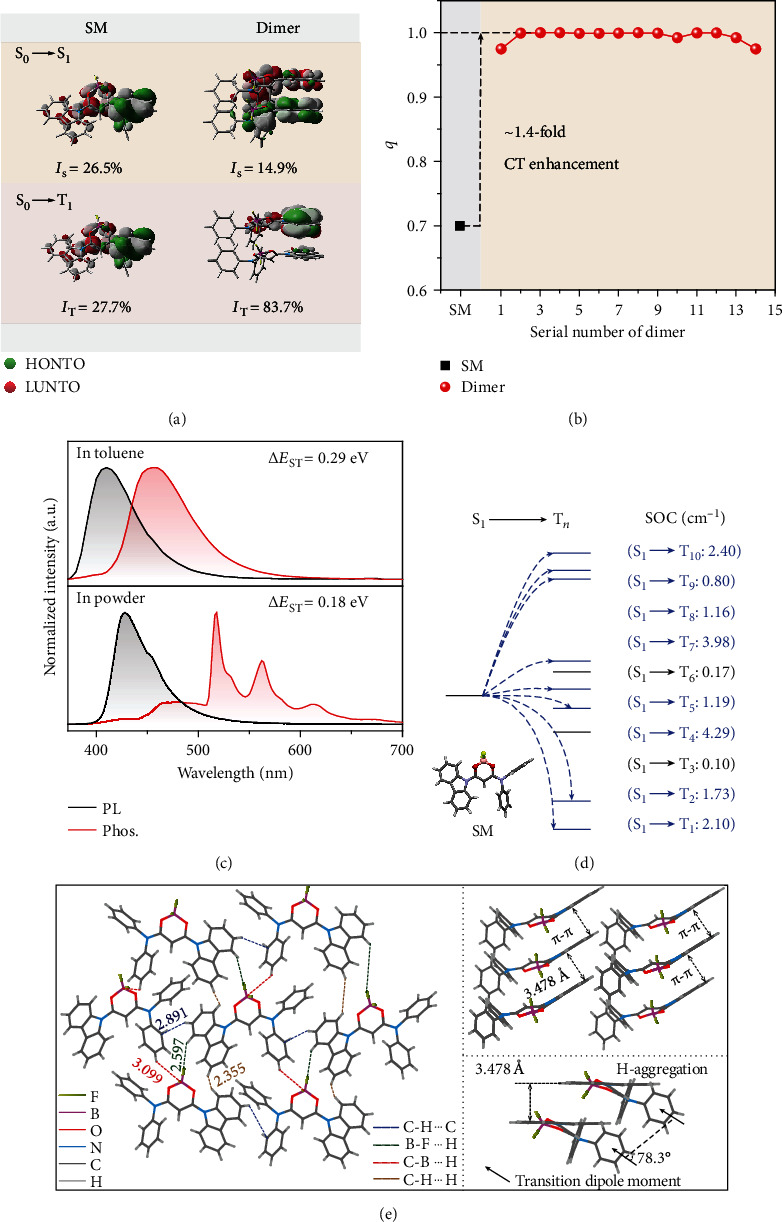
Theoretical and single-crystal analyses of CzPAB. (a) NTO analyses on S_0_ → S_1_ and S_0_ → T_1_ excitations and orbital overlap extents (*I*_S_ and *I*_T_) at single molecular (SM) and dimer states. (b) CT amount (*q*) of SM and dimers extracted from the single crystal at the S_0_ state. (c) SSPL and phosphorescent (delay 5 ms) spectra in toluene (top) and powder (bottom) at 77 K. (d) TD-DFT-calculated excited state energy levels and the SOC constants between S_1_ and T_*n*_. (e) Molecular arrangement in single crystal with various intermolecular interactions (left) and representative molecular packing (top right) for H-aggregation (bottom right).

To gain deep insights into the extraordinary MPA-OURTP feature of CzPAB powder, density functional theory (DFT) and time-dependent DFT (TD-DFT) calculations were performed to investigate the electronic structures of the excited states on both the single molecular (SM) and aggregated dimer states. Natural transition orbital (NTO) analyses show the separation of the highest occupied NTO (HONTO) and the lowest unoccupied NTO (LUNTO) isosurface at both S_1_ and T_1_ states with small frontier orbital overlap integrals (*I*_S_ and *I*_T_ < 28%) because of the strong ICT feature of CzPAB in the SM state (Figure 3(a)) [39]. Also, an apparent ICT character was observed at S_0_ in the SM state with large CT amount (*q*) over 0.7 and became more obvious in the aggregated dimer structures with ~1.4-fold enhancement of *q* [40] (Figure 3(b)) and more separated NTO distribution at S_1_ with smaller *I*_S_ with the aid of the additional SCT effect (Figure 3(a) and Figure S14). Extraordinarily, *I*_T_ of the dimer is significantly increased, which would be beneficial for the highly efficient phosphorescent emission. The theoretically predicted CT character was confirmed by both the broad structureless absorption band in dichloromethane (DCM) solution (Figure S15) and bathochromic shifted PL peaks at the increased solvent polarity (Figure S16). Again, owing to the CT nature, CzPAB exhibits a small singlet-triplet splitting energy (Δ*E*_ST_) of 0.29 eV in solution and 0.18 eV in powder estimated from the fluorescence and phosphorescence spectra at a cryogenic temperature of 77 K (Figure 3(c)). The reduced Δ*E*_ST_ in powder should be due to the enhanced CT properties by the synergistic effects of ICT and SCT in the aggregated state. These small values of Δ*E*_ST_ suggest that CzPAB should have facile ISC to populate T_1_ and reverse ISC (RISC) to return to S_1_ for efficient thermally activated delayed fluorescence (TADF), which was experimentally observed on the OURTP spectra around 430 nm (Figure 2(a)) and theoretically confirmed by the Dolton simulations with the large spin orbital coupling (SOC) values between S_1_ and T_*n*_ (Figure 3(d) and Table S2) [7, 41]. The TADF-featured OURTP have greatly improved luminescent efficiency, since the spin-forbidden triplet state emission is transformed to the spin-allowed emission of the singlet excited state by the RISC process (Figure S17). Therefore, the OURTP quantum efficiency of CzPAB powder reaches 37%, which is among the best results reported so far.

Besides the strong CT feature for the nonlinear MPA and small Δ*E*_ST_ for facile ISC to populate T_1_, H-aggregations are also crucial in realizing the MPA-OURTP [42–44]. From Figure 3(e), many H-aggregations with positive exciton splitting energies and strong *π*-*π* interaction to stabilize the triplet excitons for OURTP emission were identified in CzPAB crystal by the Frenkel exciton theory (Table S4). Moreover, the central CzPAB is surrounded by six other molecules, exhibiting strong and abundant intermolecular interactions of C-H••••C, C-B••••H, B-F••••H, and C-H••••H with corresponding distances of 2.891, 3.099, 2.597, and 2.355 Å, respectively (Figure 3(e), left). These strong intermolecular interactions can not only restrict the molecular vibration to suppress the nonradiative decays for the highly efficient emission but also provide solid evidence for the existence of SCT to reinforce the MPA ability in aggregated states.

### 2.3. Mechanism of MPA-OURTP

**Figure 4:**
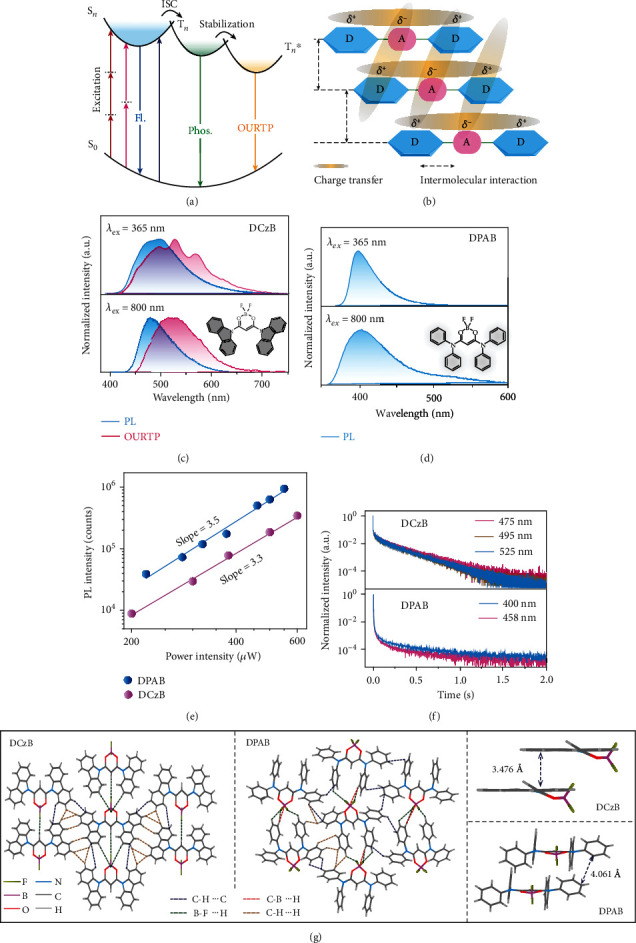
Proposed mechanism of MPA-OURTP and photophysical properties and aggregation structures of DCzB and DPAB powders. (a) Mechanisms of OURTP and MPA-OURTP. (b) Design principles of MPA-OURTP molecules by integrating intra- and intermolecular CT characters into H-aggregations in a quadrupolar D-A-D skeleton. (c, d) SSPL (blue) and OURTP (pink) spectra of the (c) DCzB and (d) DPAB powders excited by 365 nm UV light and 800 nm NIR laser. Insets show the corresponding molecular structures. (e) The logarithmic plots of the integrated emission intensity versus the 800 nm laser power. (f) OURTP lifetime decay profiles at 475, 495, and 525 nm for DCzB (top) and at 400 and 458 nm for DPAB (bottom) excited by 365 nm UV light. (g) Molecular arrangements in single crystals of DCzB (left) and DPAB (middle) with representative molecular packing structures.

Based on these experimental and theoretical findings, a possible mechanism for MPA-OURTP is proposed (Figure 4(a)). The D-A-D molecule with synergistic ICT and SCT effects in the solid state (Figure 4(b)) enables nonlinear MPA processes for NIR laser excitation to populate the singlet excited states, which transforms facilely to triplet ones by heteroatom facilitated ISC rates; the transformed triplet excitons are then stabilized by H-aggregation and intermolecular interactions to slow down or suppress both the radiative and nonradiative decays (Figure 4(b)), resulting in the efficient MPA-OURTP from the radiative decay of the stabilized triplet excitons.

To confirm the above understandings in designing MPA-OURTP molecules, we further prepared two BF_2_bdk-based derivatives in a D-A-D molecular skeleton, namely, difluoroboron 1,3-di(9H-carbazol-9-yl) propane-1, 3-dione (DCzB) and difluoroboron N^1^,N^1^,N^3^,N^3^-tetraphenylmalonamide (DPAB). Strong CT absorbance and emission peaks can be found in DCzB and DPAB solutions (Figures S18 and S19) due to the directly connected donor and acceptor units in the D-A-D architecture. Therefore, both DCzB and DPAB powders exhibit intense MPA-motivated fluorescence that is almost identical to their corresponding UV-triggered emission (Figures 4(c)–4(e)). For DCzB, obvious OURTP and MPA-OURTP were observed with lifetimes up to ~230 ms, when excited by both UV and NIR light (Figure 4(f), top panel). However, for DPAB, the phosphorescence lifetimes are only ~58 ms (Figure 4(f), bottom panel, and Figure S20). To understand the different photophysical properties of DCzB and DPAB, the single crystal structures of these two molecules were systematically investigated. Both DCzB and DPAB crystals display plenty of intermolecular interactions for the efficient SCT to facilitate the MPA process and suppress nonradiative decays, but compared to DCzB crystal with strong *π*-*π* interaction (3.476 Å) for H-aggregations, DPAB exhibits loose *π*-*π* interaction and J-aggregation dominates its solid state (Table S4). Therefore, no OURTP was observed in DPAB crystal, although its phosphorescence is quite strong with a high efficiency of ~31%. It is clear that efficient CT, facile ISC, H-aggregation, and abundant intermolecular interactions are essential for efficient MPA-OURTP.

### 2.4. Applications of MPA-OURTP Materials

**Figure 5:**
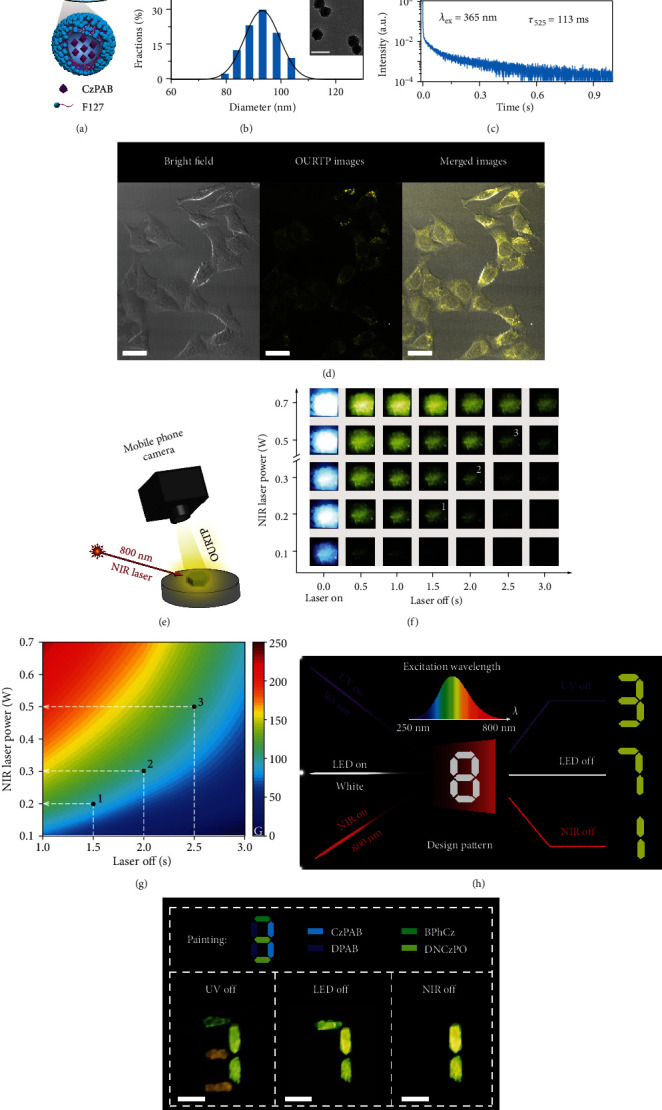
Applications of MPA-OURTP materials. (a) Schematic drawing of the bottom-up strategy to prepare CzPAB nanoparticles. (b) Particle size distribution revealed by dynamic light scattering. The inset is a transmission electron microscope image. The scale bar is 200 nm. (c) OURTP lifetime decay profile at 530 nm of CzPAB nanoparticles excited by 365 nm UV light under ambient conditions. (d) Two-photon confocal laser scanning microscopy imaging of HeLa cells stained with CzPAB nanoparticles after incubation for 6 h. The cellular images were captured by collecting the luminescence from 500 to 750 nm under the excitation of 800 nm NIR laser. The scale bar is 10 *μ*m. (e) The setup for the visual NIR laser power detector. (f) Power-dependent OURTP images of CzPAB powder from the video recorded after turning off the 800 nm NIR laser. (g) Evolution mapping of grayscale (G) values of the OURTP images under different laser off-time. (h) Design and (i) demonstration of the excitation and lifetime multiplexing encryption device. The scale bar is 1 cm.

In light of the extraordinary linear and nonlinear photoexcitation feature of the MPA-OURTP materials, we tested the multifunctional applications of CzPAB. Firstly, water-dispersible CzPAB nanoparticles with an average size of ~90-100 nm and obvious fluorescence showing excellent photostabilities and OURTP emission with lifetime of ~113 ms were prepared by the typical bottom-up approach using an amphiphilic copolymer (PEG-b-PPG-b-PEG, F127) [45] (Figures 5(a)–5(c) and Figures S21 and S22). Two-photon confocal laser scanning microscopy images (Figure 5(d)) show that CzPAB nanoparticles can facilely permeate the cytoplasm of HeLa cells and emit strong MPA-OURTP with a high signal-to-noise ratio of ~17 by the 800 nm femtosecond laser irradiation, demonstrating the great potential of MPA-OURTP materials in acquiring deep-tissue and high-resolution bioimaging. Secondly, inspired by the MPA-triggered OURTP properties, we developed a novel visual NIR laser power detector (Figure 5(e)). From the OURTP photographs extracted from the video of CzPAB powder taken by a commercial mobile phone, the OURTP intensity is closely dependent on the laser powers (Figure 5(f) and Figure S23), and their G values (Figure 5(g) and Figure S24) are quantitatively related to the excitation laser power and laser off-time (Supporting Information, Sections S9 and S10). Therefore, the NIR laser powers can be obtained by correlating the G value and laser off-time conveniently (Figure 5(g)). For instance, with the visual G values of 98, 103, and 108 at laser off-time of 1.5, 2.0, and 2.5 s located at 1, 2, and 3 in Figures 5(f) and 5(g), the excitation laser powers can be detected to be 0.2, 0.3, and 0.5 W, respectively. Thirdly, we fabricated a new encryption device (Figure 5(h)) using DPAB; the UV, LED, and NIR light-excitable OURTP molecule of CzPAB; and other normal OURTP materials (Figure S25). With different excitation sources of handheld UV light, LED flashlight, and NIR laser, the lifetime-encrypted pattern varies from “3” and “7” to “1” (Figure 5(i)) correspondingly after switching off these excitation sources, demonstrating an interesting excitation and lifetime multiplexing feature of the anticounterfeiting device.

## 3. Discussion

In conclusion, we have proposed a rational molecular design strategy of upconverted OURTP materials to enable NIR-excitable OURTP in small organic molecules with a quadrupolar D-A-D molecular skeleton. This strategy leans upon the insertion of ICT and SCT into H-aggregation to render efficient MPA ability and stabilized triplet excitons in solid states for MPA-OURTP emission. The OURTP quantum yields and lifetimes reach up to 37% and 423 ms, respectively. On account of the high-performance MPA-OURTP, two-photon-excited OURTP bioimaging, NIR laser power sensing, excitation and lifetime multiplexing encryption devices were applicable, illustrating a bright future of advanced applications with the nonlinear and NIR-excitable OURTP. We envision that the discovery of MPA-OURTP would stimulate intensive investigations on the nonlinear aspect of organic phosphors, providing a deep insight into the designing of rich upconverted photonic properties of OURTP for advanced and multifunctional device applications.

## 4. Materials and Methods

### 4.1. Preparation and Characterization of CzPAB

To a 50 mL double-neck bottle charged with 9H-carbazole (0.5 g, 3.0 mmol) and diphenylamine (0.5 g, 3.0 mmol) was injected 30 mL DCM using a syringe under an argon atmosphere. Then, the malonyl dichloride (0.3 mL, 3.0 mmol) was injected to the reaction system slowly. After stirring at room temperature for 3 hours, a BF_3_·Et_2_O (46.5% BF_3_, 1.2 mL, 9.0 mmol) solution was added into the reaction mixture slowly. To complete the reaction, the mixture was refluxed overnight. The reaction mixture was quenched with 10 mL water and extracted with DCM for three times (3 × 100mL). The organic layers were collected, combined, and dried with anhydrous sodium sulfate (Na_2_SO_4_). The solvent was removed under reduced pressure, and the residue was purified by column chromatography (silica gel, 3 : 1 *v*/*v*, petroleum ether/DCM). Yield: 0.35 g of white powder (30%). ^1^H NMR (400 MHz, d-DMSO, ppm): *δ* 8.22 (d, *J* = 7.5Hz, 2H), 7.86 (d, *J* = 7.7Hz, 2H), 7.76 (d, *J* = 8.3Hz, 2H), 7.71-7.38 (m, 12H), 5.34 (s, 1H). ^13^C NMR (100 MHz, CDCl_3_) *δ* 169.3, 164.7, 138.0, 130.4, 129.5, 128.0, 127.1, 126.4, 126.3, 123.7, 120.1, 114.8, 77.6. HRMS (ESI): *m*/*z* calcd. for C_27_H_20_BF_2_N_2_O_2_ [M + H]^+^, 453.1586; found, 453.1950.

### 4.2. Photophysical Measurements

Ultraviolet/visible (UV/Vis) absorption and photoluminescence (PL) spectra were recorded on a Jasco V-750 spectrophotometer and Edinburgh FLS980 spectrophotometer, respectively. The absolute photoluminescence quantum yield (PLQY) was obtained using an Edinburgh FLS980 fluorescence spectrophotometer equipped with an integrating sphere. For fluorescence decay measurements, a picosecond pulsed light-emitting diode (EPLED-380, wavelength: 377 nm; pulse width: 947.7 ps) was used. Phosphorescence spectra were obtained using an Edinburgh FLS980 fluorescence spectrophotometer at 77 K with a 10 ms delay time after excitation using a microsecond flash lamp. The microsecond flash lamp produces short, typically a few *μ*s, and high irradiance optical pulses for phosphorescence decay measurements in the range from microseconds to seconds. The kinetic measurements, OURTP spectra, and ultralong lifetime in powders were also measured on an Edinburgh FLS980 fluorescence spectrophotometer. For femtosecond optical spectroscopy, the laser source was a Coherent Legend regenerative amplifier (150 fs, 1 kHz, 800 nm) seeded with a Coherent Vitesse oscillator (100 fs, 80 MHz). 800 nm wavelength laser pulses were from the regenerative amplifier's output. 720 nm laser pulses with pulse width ∼50 fs were generated from an optical parametric amplifier (OperASolo) coupled to a one-box integrated Ti-Sapphire amplifier (Libra, Coherent). The emission from the samples was collected at a backscattering angle of 150° by a pair of lenses and into an optical fiber that is coupled to a spectrometer (Acton, Spectra Pro 2500i) to be detected by a charge-coupled device (Princeton Instruments, Pixis 400B). The laser pulse (circular spot, diameter 1 mm) is directly incident to the samples.

### 4.3. Theoretical Calculations

Density functional theory (DFT) and time-dependent DFT (TD-DFT) calculations were performed using the Gaussian 09 package. The ground-state geometries were optimized by the DFT method of the Lee-Yang-Parr correlation functional (B3LYP) using 6-31G(d) basis sets. The optimized static point was further carried out by harmonic vibration frequency analysis to guarantee that the real local minimum was achieved. The Dalton program with a quadratic response function method was used to predict spin-orbit coupling (SOC) matrix elements between the lowest singlet excited state (S_1_) and the lowest triplet excited state (T_1_). The SOC values were carried out on the basis of the optimized geometry of T_1_ using the B3LYP functional and 6-31G(d) basis set. Natural transition orbital (NTO) analysis was performed to get insights into the whole picture of the excited states with a compact orbital representation for the electronic transition density matrix. The overlap integrals between the highest occupied NTO (HONTO) and the lowest unoccupied NTO (LUNTO) at S_1_ (*I*_S_) and T_1_ (*I*_T_) states were also calculated using Multiwfn to take full considerations of electron transition components at the corresponding excited states.

For investigations on humans, a statement must be included indicating that informed consent was obtained after the nature and possible consequences of the study were explained.

## Supplementary Materials

Supplementary Materials Scheme S1: a survey of representative single-component organic ultralong room-temperature phosphorescence molecules. Scheme S2: synthesis of MPA-OURTP molecules. Figure S1: ^1^H NMR spectrum of CzPAB in d-DMSO. Figure S2: ^13^C NMR spectrum of CzPAB in CDCl3. Figure S3: HRMS of CzPAB. Figure S4: ^13^H NMR spectrum of DCzB in CDCl3. Figure S5: ^13^C NMR spectrum of DCzB in CDCl3. Figure S6: HRMS of DCzB. Figure S7: ^1^H NMR spectrum of DPAB in d-DMSO. Figure S8: ^13^C NMR spectrum of DPAB in CDCl3. Figure S9: TGA curves of CzPAB, DCzB, and DPAB. Figure S10: fluorescence decay profile of CzPAB powder at 300 K. Figure S11: OURTP decay profiles of CzPAB powder at 300 K. Figure S12: excitation spectrum of CzPAB by monitoring the emission peak at 530 nm and the luminescence spectrum of an iPhone 6's light-emitting diode (LED) light. Figure S13: photoluminescence intensity evolution under different excitation intensities and duration. Figure S14: molecular configuration of CzPAB dimers extracted from single crystals. Figure S15: photophysical properties in DCM solution. Figure S16: PL spectra of CzPAB in different solvents at room temperature. Figure S17: evolution of OURTP properties under different temperatures. Figure S18: absorption and fluorescence spectra of DCzB and DPAB in DCM solution. Figure S19: PL spectra of DCzB and DPAB in different solvents at room temperature. Figure S20: steady-state PL and phosphorescence spectra of DPAB crystal at 300 K. Figure S21: PL properties of NPs. Figure S22: photostabilities of NPs. Figure S23: power-dependent OURTP images of CzPAB powder from the video recorded after turning off the 800 nm NIR laser. Figure S24: the G values versus incident laser power at different laser off-time. Figure S25: molecular structures and photophysical properties of OURTP powder. Table S1: PL lifetimes of MPA-OURTP powders excited at 395 nm under ambient conditions Table S2: TD-DFT-calculated excited state energy levels and the spin-orbit coupling (SOC) constants between S_1_ and T_*n*_ of CzPAB. Table S3: crystallographic data of CzPAB, DCzB, and DPAB. Table S4: aggregation structures in CzPAB, DCzB, and DPAB crystals identified by the exciton splitting energy (Δ*ε*). Movie S1: the CzPAB crystal excited by a 365 UV lamp, LED flashlight, and 800 nm NIR laser. Movie S2: the CzPAB crystal excited by 800 nm NIR laser under different excitations.
