# Supplementary material for: Near-Infrared-Excitable Organic Ultralong Phosphorescence through Multiphoton Absorption
Source: Research (Wash D C). 2020 Dec 1;2020:2904928. doi: 10.34133/2020/2904928 (PMC7877391; doi:10.34133/2020/2904928)
Supplement: Supplementary Materials — Scheme S1: a survey of representative single-component organic ultralong room-temperature phosphorescence molecules. Scheme S2: synthesis of MPA-OURTP molecules. Figure S1: 1H NMR spectrum of CzPAB in d-DMSO. Figure S2: 13C NMR spectrum of CzPAB in CDCl3. Figure S3: HRMS of CzPAB. Figure S4: 13H NMR spectrum of DCzB in CDCl3. Figure S5: 13C NMR spectrum of DCzB in CDCl3. Figure S6: HRMS of DCzB. Figure S7: 1H NMR spectrum of DPAB in d-DMSO. Figure S8: 13C NMR spectrum of DPAB in CDCl3. Figure S9: TGA curves of CzPAB, DCzB, and DPAB. Figure S10: fluorescence decay profile of CzPAB powder at 300 K. Figure S11: OURTP decay profiles of CzPAB powder at 300 K. Figure S12: excitation spectrum of CzPAB by monitoring the emission peak at 530 nm and the luminescence spectrum of an iPhone 6's light-emitting diode (LED) light. Figure S13: photoluminescence intensity evolution under different excitation intensities and duration. Figure S14: molecular configuration of CzPAB dimers extracted from single crystals. Figure S15: photophysical properties in DCM solution. Figure S16: PL spectra of CzPAB in different solvents at room temperature. Figure S17: evolution of OURTP properties under different temperatures. Figure S18: absorption and fluorescence spectra of DCzB and DPAB in DCM solution. Figure S19: PL spectra of DCzB and DPAB in different solvents at room temperature. Figure S20: steady-state PL and phosphorescence spectra of DPAB crystal at 300 K. Figure S21: PL properties of NPs. Figure S22: photostabilities of NPs. Figure S23: power-dependent OURTP images of CzPAB powder from the video recorded after turning off the 800 nm NIR laser. Figure S24: the G values versus incident laser power at different laser off-time. Figure S25: molecular structures and photophysical properties of OURTP powder. Table S1: PL lifetimes of MPA-OURTP powders excited at 395 nm under ambient conditions Table S2: TD-DFT-calculated excited state energy levels and the spin-orbit coupling (SOC) constant [file 2904928.f1.zip › 2904928.f1/Supplementary Information-clean.doc]

Supplementary Information

**Near-Infrared Excitable Organic Ultralong Phosphorescence through Multiphoton Absorption**

Ye Tao, Lele Tang, Qi Wei, Jibiao Jin, Wenbo Hu, Runfeng Chen,* Qingqing Yang, Huanhuan Li, Ping Li, Guichuan Xing, Quli Fan,* Chao Zheng, Wei Huang*

**Content**

1. Background information	S2

2. Synthesis and characterization	S3

3. Thermal properties	S8

4. Photophysical investigations	S9

5. Theoretical calculations	S14

6. Charge transfer (CT) properties of CzPAB	S16

7. Single crystal structure analysis	S18

8. Photoluminescent investigations of DCzB and DPAB	S21

9. Nonlinear optoelectronic applications	S22

10. Video recording of MPA-OURTP	S27

11. References	S28

### 1. Background information

**Scheme S1**. **A survey of representative single-component organic ultralong room temperature phosphorescence molecules.** The representative single-component organic ultralong room temperature phosphorescence materials with corresponding maximum excitation wavelength (λ_Ex_), main emission peak (λ_Em_), Stokes-shift, lifetimes (τ), and phosphorescent yield (QY)^[1-7]^.

### 2. Synthesis and characterization

**Materials:** All reagents, unless otherwise specified, were purchased from Aldrich, Acros or Alfa Aesar, and used without further purification. Manipulations involving air-sensitive reagents were performed in an atmosphere of dry argon. Dichloromethane (DCM) was dried and purified by routine procedures.

**Instruments:** ^1^H and ^13^C-nuclear magnetic resonance (NMR) spectra were recorded on a Bruker Ultra Shied Plus 400 MHz instrument with CDCl_3_ and *d*-DMSO as the solvents and tetramethylsilane (TMS) as the internal standard. High resolution mass spectra (HRMS) were collected by a LCT Premier XE (Waters) HRMS spectrometry.

**Scheme S2**. **Synthesis of MPA-OURTP molecules.** Synthesis of **CzPAB**, **DCzB** and **DPAB**. Conditions: (i) 9*H*-carbazole, or diphenylamine, DCM, room temperature, 3 h; (ii) BF_3_·Et_2_O, 40ºC, 12 h.

**Difluoroboron 3-(9*H*-carbazol-9-yl)-3-oxo-N,N-diphenylpropanamide (CzPAB)**

To a 50 mL double-neck bottle charged with 9*H*-carbazole (0.5 g, 3.0 mmol) and diphenylamine (0.5 g, 3.0 mmol) was injected 30 mL DCM using a syringe under an argon atmosphere. Then, the malonyl dichloride (0.3 mL, 3.0 mmol) was injected to the reaction system slowly. After stirring at room temperature for 3 hours, a BF_3_·Et_2_O (46.5% BF_3_, 1.2 mL, 9.0 mmol) solution was added into the reaction mixture slowly. To complete the reaction, the mixture was refluxed overnight. The reaction mixture was quenched by 10 mL water, and extracted with DCM for three times (3×100 mL). The organic layers were collected, combined and dried with anhydrous sodium sulfate (Na_2_SO_4_). The solvent was removed under reduced pressure and the residue was purified by column chromatography (silica gel, 3:1 v/v, petroleum ether/ DCM). Yield: 0.35 g of white powder (30%). ^1^H NMR (400 MHz, *d*-DMSO, ppm): δ 8.22 (d, J=7.5 Hz, 2H), 7.86 (d, J=7.7 Hz, 2H), 7.76 (d, J=8.3 Hz, 2H), 7.71-7.38 (m, **12H**), 5.34 (s, 1H). ^13^C NMR (100 MHz, CDCl_3_) δ 169.3, 164.7, 138.0, 130.4, 129.5, 128.0, 127.1, 126.4, 126.3, 123.7, 120.1, 114.8, 77.6. HRMS (ESI): *m/z* calcd. for C_27_H_20_BF_2_N_2_O_2_ [M+H]^+^, 453.1586; found, 453.1950.

**Difluoroboron 1, 3-Di(9*H*-carbazol-9-yl)propane-1, 3-dione (DCzB)**

**DCzB** was prepared under the identical synthetic conditions described in the preparation of **CzPAB**, using 9*H*-carbazole (1.0 g, 6.0 mmol), malonyl dichloride (0.3 mL, 3.0 mmol), and BF_3_‧Et_2_O (46.5% BF_3_, 1.2 mL, 9.0 mmol). Yield: 0.70 g of green powder (60%). ^1^H NMR (400 MHz, CDCl_3_): δ 8.24 (d, J = 8.0 Hz, 4H), 8.05 (d, J = 8.0 Hz, 4H), 7.57 (t, J = 16 Hz, 4H), 7.48 (t, J = 16 Hz, 4H), 6.79 (s, 1H). ^13^C NMR (100 MHz, CDCl_3_): δ 167.7, 137.86, 127.88, 127.2, 125.13, 120.57, 115.89, 80.27. HRMS (ESI): *m/z* calcd. for C_27_H_18_BF_2_N_2_O_2_ [M+H]^+^, 451.1429; found, 451.1437.

**Difluoroboron** **N^1^,N^1^,N^3^,N^3^-tetraphenylmalonamide (DPAB)**

**DPAB** was prepared under the identical synthetic conditions described in the preparation of **CzPAB**, using diphenylamine (1.0 g, 6.0 mmol), malonyl dichloride (0.3 mL, 3.0 mmol), and BF_3_·Et_2_O (46.5% BF_3_, 1.2 mL, 9.0 mmol). Yield: 0.92 g of white powder (80%).^1^H NMR (400 MHz, *d*-DMSO, ppm): δ 7.40 (t, *J* = 7.7 Hz, 8H), 7.36-7.25 (m, 12H), 4.03 (s, 1H). ^13^C NMR (100 MHz, CDCl_3_) δ 167.6, 141.2, 129.3, 127.3, 127.2, 71.3. HRMS (ESI): *m/z* calcd. for C_27_H_22_BF_2_N_2_O_2_ [M+H]^+^, 455.1737; found, 451.1746.

**Fig. S1**. ^1^H NMR spectrum of **CzPAB** in *d*-DMSO.

**Fig. S2**. ^13^C NMR spectrum of **CzPAB** in CDCl_3_.

**Fig. S3**. HRMS of **CzPAB**.

**Fig. S4**. ^13^H NMR spectrum of **DCzB** in CDCl_3_.

**Fig. S5**. ^13^C NMR spectrum of **DCzB** in CDCl_3_.

**Fig. S6**. HRMS of **DCzB**.

**Fig. S7**. ^1^H NMR spectrum of **DPAB** in *d*-DMSO.

**Fig. S8**. ^13^C NMR spectrum of **DPAB** in CDCl_3_.

### 3. Thermal properties

Thermogravimetric analyses (TGA) were conducted on a DTG-60 Shimadzu thermal analyst system under a heating rate of 10ºC/min and a nitrogen flow rate of 50 cm^3^/min. The differential scanning calorimetry (DSC) analyses were performed on a Shimadzu DSC-60A instrument under a heating rate of 10ºC/min and a nitrogen flow rate of 20 cm^3^/min.

**Fig. S9.** TGA curves of **CzPAB**, **DCzB**, and **DPAB**.

### 4. Photophysical investigations

Ultraviolet/visible (UV/Vis) absorption and photoluminescence (PL) spectra were recorded on a Jasco V-750 spectrophotometer and Edinburgh FLS980 spectrophotometer, respectively. The absolute photoluminescence quantum yield (PLQY) was obtained using an Edinburgh FLS980 fluorescence spectrophotometer equipped with an integrating sphere. For fluorescence decay measurements, a picosecond pulsed light emitting diode (EPLED-380, wavelength: 377 nm; pulse width: 947.7 ps) was used. Phosphorescence spectra were obtained using an Edinburgh FLS980 fluorescence spectrophotometer at 77 K with a 10 ms delay time after excitation using a microsecond flash lamp. The microsecond flash lamp produces short, typically a few μs, and high irradiance optical pulses for phosphorescence decay measurements in the range from microseconds to seconds. The kinetic measurements, OURTP spectra, ultralong lifetime in powders were also measured on an Edinburgh FLS980 fluorescence spectrophotometer. The lifetimes (*τ*) of the luminescence were obtained by fitting the decay curve with a multi-exponential decay function of

where *A_i_* and *τ_i_* represent the amplitudes and lifetimes of the individual components for multi-exponential decay profiles, respectively. For femtosecond optical spectroscopy, the laser source was a Coherent Legend regenerative ampliﬁer (150 fs, 1 kHz, 800 nm) seeded by a Coherent Vitesse oscillator (100 fs, 80 MHz). 800 nm wavelength laser pulses were from the regenerative ampliﬁer’s output. 720 nm laser pulses with pulse width ∼50 fs was generated from an optical parametric amplifier (OperASolo) coupled to a one-box integrated Ti-Sapphire amplifier (Libra, Coherent). The emission from the samples was collected at a backscattering angle of 150° by a pair of lenses and into an optical ﬁber that is coupled to a spectrometer (Acton, Spectra Pro 2500i) to be detected by a charge coupled device (Princeton Instruments, Pixis 400B). The laser pulse (circular spot, diameter 1 mm) is directly incident to the samples.

**Fig. S10.** Fluorescence (430 nm) decay profile of **CzPAB** powder at 300 K. The excitation wavelength is 395 nm.

**Fig. S11.** OURTP decay profiles of **CzPAB** powder at 300 K. The emission peaks are 430, 455, and 530 nm, while the excitation wavelength is 395 nm.

**Fig. S12.** Excitation spectrum of **CzPAB** by monitoring the emission peak at 530 nm and the luminescence spectrum of an iPhone 6’s light emitting diode (LED) light.

To systemically investigate the influence of excitation intensity and duration on the OURTP properties, kinetic analyses were performed. The excitation intensity is regulated by tuning the Iris aperture of Edinburgh FLS980 from 20% to 100%. In **Figure S13a, b**, the excitation light at 395 nm with varied Iris was turned on and maintained for 10 s, and then turned off. Both of steady-state and OURTP emission were strengthened with the increasing of excitation intensity. Notably, the OURTP emission remains stable during the excitation and then decay in exponential manner after turned-off the excitation light, demonstrating clearly its ultralong lifetime feature. In **Figure S13c, d,** the influence of excitation duration on the OURTP emission was studied by measuring its intensity by varying irradiation duration time. The steady-state and OURTP emission intensities increase rapidly and become almost constant when the excitation duration varies from 0.1 to 10 s. These results indicate that the OURTP emission can be effectively motivated by low intensity and short duration of incident UV light under ambient conditions.

**Fig. S13.** **Photoluminescence intensity evolution under different excitation intensity and duration.** (**a**) Photoluminescence (PL) intensity profiles of 530 nm for **CzPAB** powder with (**b**) steady-state PL (black) and OURTP (red) intensities as a function of time and Iris (20, 40, 60, 80 and 100%) excited at 395 nm under ambient conditions. (**c**) PL intensity profiles of 530 nm for **CzPAB** powder with (**d**) corresponding steady-state PL (black) and OURTP (red) intensities as a function of time upon 395 nm excitation with different irradiating time (0.1, 0.4, 1.0, 2.0, 4.0, 8.0, 10 s) under ambient conditions.

**Table S1**. PL lifetimes of **CzPAB,** **DCzB**, and **DPAB** powders excited at 395 nm under ambient conditions

|  | Wavelength (nm) | τ_1_ (ms) | A_1_ (%) | τ_2_ (ms) | A_2_ (%) | τ_3_ (ms) | A_3_ (%) | χ^2^ |
| --- | --- | --- | --- | --- | --- | --- | --- | --- |
| **CzPAB** | 430 | 34.9 | 39.97 | 273.3 | 30.3 | 1016.4 | 29.72 | 1.181 |
|  | 455 | 34.4 | 39.82 | 264.9 | 30.18 | 988 | 30.00 | 1.138 |
|  | 530 | 393 | 100 |  |  |  |  | 1.048 |
|  | 575 | 383 | 100 |  |  |  |  | 1.276 |
|  | 625 | 371 | 100 |  |  |  |  | 1.070 |
| **DCzB** | 475 | 41.75 | 18.05 | 240.12 | 81.95 |  |  | 1.138 |
|  | 495 | 37.38 | 18.76 | 234.39 | 81.24 |  |  | 1.271 |
|  | 525 | 36.18 | 18.39 | 231.98 | 81.61 |  |  | 1.288 |
| **DPAB** | 400 | 2.00 | 19.89 | 17.83 | 34.95 | 103.86 | 45.16 | 1.298 |
|  | 458 | 0.7 | 27.19 | 5.9 | 35.16 | 53.01 | 37.65 | 1.216 |

### 5. Theoretical calculations

Density functional theory (DFT) and time-dependent DFT (TD-DFT) calculations were performed using Gaussian 09 package. The ground state geometries were optimized by DFT method of the Lee–Yang–Parr correlation functional (B3LYP) using 6-31G(d) basis sets. The optimized static point was further carried out by harmonic vibration frequency analysis to guarantee that the real local minimum was achieved. Dalton program with quadratic response function method was used to predict spin-orbit coupling (SOC) matrix elements between the lowest singlet excited state (S_1_) and the lowest triplet excited state (T_1_).^[8, 9]^ The SOC values were carried out on the basis of the optimized geometry of T_1_ using B3LYP functional and 6-31G(d) basis set.

Natural transition orbital (NTO) analysis was performed to get insights into the whole picture of the excited states with a compact orbital representation for the electronic transition density matrix. The overlap integrals between the highest occupied NTO (HONTO) and the lowest unoccupied NTO (LUNTO) at S_1_ (*I*_S_) and T_1_ (*I*_T_) states were also calculated using Multiwfn to take full considerations of electron transition components at the corresponding excited states.

**Fig. S14.** Molecular configuration of **CzPAB** dimers extracted from single crystals.

**Table S2**. TD-DFT calculated excited state energy levels and the spin-orbit coupling (SOC) constants between S_1_ and T_n_ of **CzPAB**.

| S_1_/T_n_ | **S_1_/T_n_ energy gap** | S_1_/Tn SOC |
| --- | --- | --- |
| S_1_/T_1_ | 0.62 | 2.10 |
| S_1_/T_2_ | 0.49 | 1.73 |
| S_1_/T_3_ | 0.17 | 0.10 |
| S_1_/T_4_ | 0.06 | 4.29 |
| S_1_/T_5_ | -0.03 | 1.19 |
| S_1_/T_6_ | -0.11 | 0.17 |
| S_1_/T_7_ | -0.16 | 3.98 |
| S_1_/T_8_ | -0.54 | 1.16 |
| S_1_/T_9_ | -0.58 | 0.80 |
| S_1_/T_10_ | -0.66 | 2.40 |

### 6. Charge transfer (CT) properties of CzPAB

**Fig. S15.** **Photophysical properties in DCM solution.** Absorption (black) and fluorescence (red) spectra of **CzPAB** in dilute (~ 10^-5^ mol L^-1^) DCM solution.

**Fig. S16.** PL spectra of **CzPAB** in different solvents (Hexane, Toluene, DCM) at room temperature (~ 10^-5^ mol L^-1^).

**Fig. S17. Evolution of OURTP properties under different temperature.** Temperature-dependent OURTP spectra (**a**) and lifetime decay profiles (**b**) of **CzPAB** powder. The emission bands are at 430, 455, and 530 nm and the excitation wavelength is 395 nm.

As shown in **Figure S17**, the room temperature OURTP spectrum exhibits a tri-mode emission with three peaks at 430, 455 and 530 nm, corresponding to the S_1_, T_1_ and stabilized T_1_ (T_1_*) states respectively. With the decrease of the temperature, the emission bands at 455 and 530 nm are remarkably enhanced with elongated lifetime owing to the suppressed non-radiative decay of excitons at low temperatures. When the temperature increases, the emission peak at 430 nm show a slightly increased intensity with elongated lifetime, due to the exciton release from the dark T^1^* to the bright S_1_.^[10]^ This is very resembling to the thermally activated delayed fluorescence (TADF) that show raised fluorescence at elevated temperature. This supernormal tri-mode OURTP emission phenomenon should be an important reason for the high PL quantum yield of **CzPAB** powder up to 37%.

### 7. Single crystal structure analysis

Single crystals of **CzPAB**, **DCzB** and **DPAB** were obtained by slow evaporation of a mixed DCM and petroleum ether solution at room temperature. Their single crystal structures were collected on a Bruker SMART APEX (II)-CCD at 296K and crystal structures were analyzed by Mercury 1.4 software. Single crystal data were summarized in **Table S3**.

**Table S3**. Crystallographic data of **CzPAB**, **DCzB** and **DPAB**.

| **Compound** | **CzPAB** | **DCzB** | **DPAB** |
| --- | --- | --- | --- |
| Empirical formula | C_27_H_19_BF_2_N_2_O_2_ | C_27_H_17_BF_2_N_2_O_2_ | C_27_H_21_BF_2_N_2_O_2_ |
| Formula weight (g mol^-1^) | 452.25 | 450.23 | 454.27 |
| Crystal color | colorless | green | colorless |
| Wavelength (Å) | 0.71073 | 0.71073 | 0.71073 |
| Crystal system | Monoclinic | Orthorhombic | Monoclinic |
| Space group | Pc | Pnma | P21/c |
| *a*, (Å) | 5.4813(11) | 14.382(16) | 5.725(3) |
| *b*, (Å) | 12.114(2) | 29.72(3) | 20.485(9) |
| *c*, (Å) | 16.743(3) | 5.070(5) | 19.617(9) |
| *α*, (deg) | 90 | 90 | 90 |
| *β*, (deg) | 96.832(5) | 90 | 90 |
| *γ*, (deg) | 90 | 90 | 90 |
| volume, (Å^3^) | 1103.8(4) | 2167(4) | 2300.6(19) |
| *Z* | 2 | 4 | 4 |
| Density, (g cm^-3^) | 1.36 | 1.38 | 1.312 |
| *μ*, (mm^-1^) | 0.097 | 0.099 | 0.0093 |
| *F* (000) | 468 | 928 | 944 |
| *h*_max_, *k*_max_, *l*_max_ | 7, 12, 21 | 14, 38, 6 | 7, 26, 26 |
| *Theta*_max_ | 27.875 | 28.965 | 28.137 |

Aggregation structures have great impact on the photophysical properties of organic optoelectronic materials. Especially, H-aggregation plays a vital role in stabilizing the excited triplet excitons for realizing the OURTP emission at the solid state. The presence of H-aggregation can be identified by Frenkel exciton theory based on the single crystal structures, where the exciton splitting energy (Δ*ε*) of dimer can be calculated by:

$$\Delta\varepsilon=\frac{2\left|M\right|^{2}}{r_{uv}^{3}}\left(\cos\alpha-3\cos\theta_{1}\cos\theta_{2}\right)$$

where *M* is the transition moment that can be simulated by TD-DFT computation, *r*_uv_ is the intermolecular distance between the selected dimer, *α* is the angle between the transition dipole moments, *θ*_1_ and *θ*_2_ are angles between transition dipole moments of the two molecules and the interconnection of their molecular centers. When Δ*ε*>0, it is H-aggregation, and when Δ*ε*<0, it belongs to J-aggregation.

**Table S4**. Aggregation structures in **CzPAB**, **DCzB** and **DPAB** crystals identified by the exciton splitting energy (Δ*ε*).

| **Compound** | **Aggregation** | **α (°)** | **θ_1_ (°)** | **θ_2_ (°)** | **M (Debye)** | **r (Å)** | **Δε (eV)** |
| --- | --- | --- | --- | --- | --- | --- | --- |
| CzPAB | H1 | 139.4 | 47.1 | 116.0 | 6.07 | 11.76 | 0.01 |
|  | H2 | 139.4 | 33.4 | 111.7 | 6.07 | 10.92 | 0.01 |
|  | H3 | 139.4 | 68.3 | 146.6 | 6.07 | 10.92 | 0.01 |
|  | H4 | 139.4 | 64.0 | 132.9 | 6.07 | 11.76 | 0.01 |
|  | H5 | 0.0 | 101.7 | 101.7 | 6.07 | 5.48 | 0.25 |
|  | H6 | 168.6 | 159.7 | 13.3 | 6.07 | 12.11 | 0.05 |
| DCzB | H1 | 0.0 | 90.00 | 90.00 | 7.53 | 5.07 | 0.54 |
|  | H2 | 0.0 | 90.00 | 90.00 | 7.53 | 5.07 | 0.54 |
|  | H3 | 180.0 | 19.74 | 160.26 | 7.53 | 15.79 | 0.03 |
|  | H4 | 180.0 | 19.74 | 160.26 | 7.53 | 15.79 | 0.03 |
|  | H5 | 180.0 | 160.26 | 19.74 | 7.53 | 15.79 | 0.03 |
|  | H6 | 180.0 | 160.26 | 19.74 | 7.53 | 15.79 | 0.03 |
| DPAB | J1 | 108.2 | 92.8 | 92.8 | 6.96 | 10.2 | -0.02 |
|  | J2 | 108.2 | 105.9 | 105.9 | 6.96 | 10.2 | -0.03 |
|  | J3 | 108.2 | 74.1 | 74.1 | 6.96 | 10.2 | -0.03 |
|  | J4 | 108.2 | 87.2 | 87.2 | 6.96 | 10.2 | -0.02 |
|  | J5 | 108.2 | 62.2 | 62.2 | 6.96 | 13.0 | -0.03 |
|  | J6 | 71.8 | 52.9 | 43.3 | 6.96 | 12.5 | -0.03 |
|  | J7 | 71.8 | 136.7 | 127.1 | 6.96 | 12.5 | -0.03 |
|  | J8 | 71.8 | 30.5 | 62.5 | 6.96 | 12.5 | -0.03 |
|  | J9 | 71.8 | 117.5 | 149.5 | 6.96 | 12.5 | -0.03 |

### 8. Photoluminescent investigations of DCzB and DPAB

**Fig. S18.** Absorption (solid symbol) and fluorescence (open symbol) spectra of **DCzB** and **DPAB** in (~ 10^-5^ mol L^-1^) DCM solution.

**Fig. S19.** PL spectra of (a) **DCzB** and (b) **DPAB** in different solvents (Hexane, Toluene, DCM) at room temperature (~ 10^-5^ mol L^-1^).

**Fig. S20.** Steady-state PL (top) and phosphorescence spectra (bottom, delay 10 ms) of **DPAB** crystal at 300 K. The excitation wavelength is 365 nm.

### 9. Nonlinear optoelectronic applications

*Preparation of water-soluble CzPAB nanoparticles (NPs)*: The **CzPAB** NPs in aqueous solution were conveniently prepared using a well-established matrix-encapsulation strategy *via* a bottom-up method (nanoprecipitation) with moderate optimization. Pluronic F127 (PEG-b-PPG-b-PEG) was selected as the matrix because of its high biocompatibility and good encapsulation performance. Generally, **CzPAB** (1 mg) and F127 (10 mg) were dissolved into a THF (1 mL) solution. Then, the mixture was rapidly injected into distilled-deionized water (9 mL) under continuous sonication with a microtip-equipped probe sonicator for 2 min. After sonication, THF was evaporated at room temperature. Finally, the aqueous solution was filtered through a 0.22 µm polyvinylidene fluoride (PVDF) syringe driven filter (Millipore).

**Fig. S21**. **PL properties of NPs.** Fluorescence (black) and OURTP spectra (red curve, delay 100 ms) of the water-soluble **CzPAB** NPs.

**Fig. S22**. **Photostabilities of NPs.** (a) The fluorescence spectra and (b) corresponding intensity of the water-soluble the water-soluble **CzPAB** nanoparticles recorded at different times.

*Characterization of CzPAB NPs*: Dynamic light scattering (DLS) and transmission electron microscope (TEM) were used to determinate the particle size and morphology of the **CzPAB** NPs. DLS was conducted using a quartz cuvette at 25 °C. Average particle sizes were characterized by laser light scattering with a particle size analyzer (90 Plus, Brookhaven Instruments Co., Holtsville, NY, USA) at a fixed angle of 90° under ambient conditions. Samples for TEM measurements were prepared by drop casting the **CzPAB** NPs dispersion onto copper grids and dried under ambient conditions, then the TEM images were obtained using a Hitachi H-H7500 microscope operated at 120 kV.

*Cell culture*: HeLa cells were cultured in in 75 cm^2^ flasks containing DMEM (Dulbecco’s Modified Eagle Medium) (GIBCO) supplemented with 10% fetal bovine serum (FBS), 1% penicillin streptomycin glutamine (GIBCO) at 37ºC in a humidified incubator of 5% CO_2_. Then, cells were carefully harvested and split when they reached a certain confluence for following use.

*Two-photon cell imaging***:** HeLa cells (4×10^3^ per well) were seeded on confocal dishes and incubated for 24 h. After that, cells were further incubated with **CzPAB** NPs (2 μg/mL) for 6 h, and then the cells were washed three times with phosphate buffer saline (PBS) for confocal imaging. Confocal laser scanning microscopy (ZEISS LSM880 NLO) equipped with femtosecond laser (Tunable range: 680-1080 nm, Pulse width: 140 fs, Repetition Rate: 80 MHz, Chameleon Ultra, Coherent) was employed to acquire two-photon induced fluorescence images. The cellular images stained with **CzPAB** NPs were captured by collecting the luminescence from 500 to 750 nm under the excitation of a femtosecond Ti: Sapphire laser at 800 nm.

**Self-designed NIR ultrafast laser power detecting**

*MPA-OURTP video recording*: The **CzPAB** powders were placed on the top of darkness filter. Under the excitation of 800 nm semiconductor ultrafast laser, the MPA motivated fluorescence and OURTP emission were captured by Nikon D90 camera.

*Grayscale* *figuring*: The MPA-OURTP video taken by commercial mobile phone (HUAWEI Meta 20 Pro) was parsed into images frame by frame using windows media player software. The red, green, and blue (RGB) values of the captured image were analyzed by the photoshop CS 6 program, and the brightness of the image could be quantized to grayscale (G) according to the following equation:^[11]^

$$Grayscale\text{ }\left(G\right)=0.2989\times Red+0.5870\times Green\text{ }+0.1140\times Blue$$

By varying the excitation power of NIR ultrafast laser, the G values versus laser power at different laser off time were obtained and plotted (**Figures 22** and **23**).

**Fig. S23**. Power-dependent OURTP images of **CzPAB** powder from the video recorded after turning off the 800 nm NIR laser.

**Fig. S24**. The G values versus incident laser power at different laser off time.

The function was fitted for power detection based on the G value of OURTP intensity of **CzPAB** powder at different laser off time. As shown in **Figure S23**, the excitation laser power of **CzPAB** range from 0.1 to 0.7 W can be verified by means of the G value of OURTP intensity in powder using the following equation:

$$\text{y = a-b}\times\text{ln(x+c)}$$

where y is G value, x represents laser power, a and b are constants. By using the fitting function, the evolution mapping of G values of the OURTP images at varied laser off time can be resulted.

**Excitation and lifetime multiplexing encryption devices**

The pattern “8” was fabricated using **CzPAB** powder in combination with phosphorescence powder of **DPAB** and OURTP powder of (9*H*-carbazol-9-yl)(phenyl)methanone^[5]^ (**BPhCz**) and di(9*H*-carbazol-9-yl)(phenyl)phosphine oxide (**DNCzPO**) (**Figure S24**).^[12]^ The OURTP emission of **CzPAB**, **BPhCz** and **DNCzPO** can be triggered by ultraviolet light. After the switch of the UV light, the OURTP pattern is 7 due to the short lifetime of **DPAB** powder. When the pattern was excited by the white light, the OURTP pattern would change to 3 after the remove of the LED flashlight because of the visible-light-excited OURTP feature of **BPhCz** and **CzPAB**. And only **CzPAB** can be pumped by 800 nm NIR laser, thus the OURTP pattern would be 1 after turning-off the NIR laser.

**Fig. S25**. **Molecular structures and photophysical properties of OURTP powder.** (a) Molecular structure of **BPhCz** and **DNCzPO**. (b) Excitation spectra of **DNCzPO** (purple) and **BPhCz** (blue) by monitoring emission peaks at 537 and 536 nm and the luminescence spectrum of an iPhone 6’s LED light.

### 10. Video recording of MPA-OURTP

The videos 1 and 2 were recorded by a Nikon D90 camera and HUAWEI Meta 20 Pro under ambient conditions, respectively.

**Video 1.** The **CzPAB** crystal excited at 365 UV lamp, LED flashlight and 800 nm NIR laser. When excited with a 365 nm hand-held UV lamp (5 W), the **CzPAB** sample emitted intense blue light. After the switch off of the UV lamp, an emission color changed from blue to yellow light was clearly observed by the naked eye. The same emission phenomenon can be clearly found by using LED flashlight and 800 nm NIR laser as the excitation source, respectively.

**Video 2.** The **CzPAB** crystal excited at 800 nm NIR laser under different excitation power.

### 11. References

[1] Z. An, C. Zheng, Y. Tao et al., "Stabilizing triplet excited states for ultralong organic phosphorescence". *Nature Materials*, vol. 14, no. 7, pp. 685-690, 2015.

[2] L. Gu, H. Shi, L. Bian et al., "Colour-tunable ultra-long organic phosphorescence of a single-component molecular crystal". *Nature Photonics*, vol. 13, no. 6, pp. 406-411, 2019.

[3] W. Zhao, T. S. Cheung, N. Jiang et al., "Boosting the efficiency of organic persistent room-temperature phosphorescence by intramolecular triplet-triplet energy transfer". *Nature Communications*, vol. 10, no. 1, pp. 1595, 2019.

[4] J. Yuan, R. Chen, X. Tang et al., "Direct population of triplet excited states through singlet–triplet transition for visible-light excitable organic afterglow". *Chemical Science*, vol. 10, no. 19, pp. 5031-5038, 2019.

[5] S. Cai, H. Shi, J. Li et al., "Visible-Light-Excited ultralong organic phosphorescence by manipulating intermolecular interactions". *Advanced Materials*, vol. 29, no. 35, article. 1701244, 2017.

[6] D. Tu, S. Cai, C. Fernandez et al., "Boron-Cluster-Enhanced ultralong organic phosphorescence". *Angewandte Chemie International Edition*, vol. 58, no. 27, pp. 9129-9133, 2019.

[7] Z. Mao, Z. Yang, C. Xu et al., "Two-photon-excited ultralong organic room temperature phosphorescence by dual-channel triplet harvesting". *Chemical Science*, vol. 10, no. 31, pp. 7352-7357, 2019.

[8] K. Aidas, C. Angeli, K. L. Bak et al., "The Dalton quantum chemistry program system". *Wiley Interdisciplinary Reviews: Computational Molecular Science*, vol. 4, no. 3, pp. 269-284, 2014.

[9] G. Baryshnikov, B. Minaev and H. Ågren, "Theory and calculation of the phosphorescence phenomenon". *Chemical Reviews*, vol. 117, no. 9, pp. 6500-6537, 2017.

[10] J. Jin, H. Jiang, Q. Yang et al., "Thermally activated triplet exciton release for highly efficient tri-mode organic afterglow". *Nature Communications*, vol. 11, no. 1, pp. 842, 2020.

[11] Y. Zhou, W. Qin, C. Du et al., "Long-Lived Room-Temperature phosphorescence for visual and quantitative detection of oxygen". *Angewandte Chemie International Edition*, vol. 58, no. 35, pp. 12102-12106, 2019.

[12] Y. Tao, R. Chen, H. Li et al., "Resonance-Activated Spin-Flipping for efficient organic ultralong Room-Temperature phosphorescence". *Advanced Materials*, vol. 30, no. 44, article. 1803856, 2018.
